# Treatment of refractory MALT lymphoma by lenalidomide plus bendamustine: A case report

**DOI:** 10.1097/MD.0000000000028938

**Published:** 2022-03-04

**Authors:** Zhencang Zhou, Pengqiang Wu, Fujue Wang, Huan Tao, Yingying Chen, Jie Gao, Dengke Chen, Yongqian Jia

**Affiliations:** a Department of Hematology and Research Laboratory of Hematology, West China Hospital of Sichuan University, Chengdu, Sichuan, China; b Department of Hematology, The Third Affiliated Hospital of Zunyi Medical University (The First People's Hospital of Zunyi ), Zunyi, Guizhou, China; c Department of Hematology, Shanghai General Hospital, Shanghai Jiao Tong University, Shanghai, China.

**Keywords:** API2/MALT, bendamustine, lenalidomide, refractory MALT lymphoma, ultrasound gastroscopy

## Abstract

**Rationale:**

Marginal zone B cell lymphoma of the mucosa-associated lymphoid tissue (MALT lymphoma) has an indolent natural course and disseminates slowly. However, there is currently no consensus regarding the optimal treatment strategy for relapsed/refractory MALT lymphomas. Lenalidomide-bendamustine may be an effective regimen for such cases.

**Patient concerns:**

A 48-year-old Chinese male patient with MALT lymphoma and API2/MALT received 2 courses of standard-dose rituximab, cyclophosphamide, vincristine, prednisone regimen chemotherapy combined with Helicobacter pylori eradication therapy. However, this disease was not effectively managed.

**Diagnosis:**

MALT lymphoma.

**Interventions:**

The patient received lenalidomide-bendamustine (lenalidomide 25 mg on days 1–21 and bendamustine 90 mg/m^2^ on days 1–2) for 6 courses.

**Outcomes:**

Lenalidomide-bendamustine was a safe and effective chemotherapy. No serious adverse events occurred during the treatment period. Ultrasound gastroscopy revealed that the tumor gradually shrank and eventually disappeared to complete remission.

**Lessons:**

The lenalidomide-bendamustine scheme might be a potentially effective option for patients with refractory or relapsed MALT lymphoma.

## 1 Introduction

Extranodal marginal zone B-cell lymphoma of the mucosa-associated lymphoid tissue (MALT lymphoma) is a special type of B-cell lymphoma characterized by its ability to reside in mucosa-associated lymphoid tissue throughout the body. It can be subdivided into gastric and non-gastric cases.^[1]^ Antibiotic eradication of Helicobacter pylori (HP) has become an effective therapy for patients with gastric MALT lymphoma, and the disease usually has a favorable prognosis.^[2]^ However, API2/MALT1-positive MALT lymphoma in the stomach is often refractory to HP eradication therapy, and has a significantly higher recurrence rate.^[3]^

Currently, there is no consensus on the optimal management of patients with multifocal, HP-negative, or persistent disease after HP eradication.^[4]^ In this report, we describe a case of MALT lymphoma with API2/MALT refractory to HP eradication therapy. We observed that the disease continued to progress after 2 cycles of rituximab, cyclophosphamide, vincristine, prednisone (R-CVP) chemotherapy but could be successfully managed and controlled with lenalidomide and bendamustine.

## 2 Case presentation

**Figure 1:**
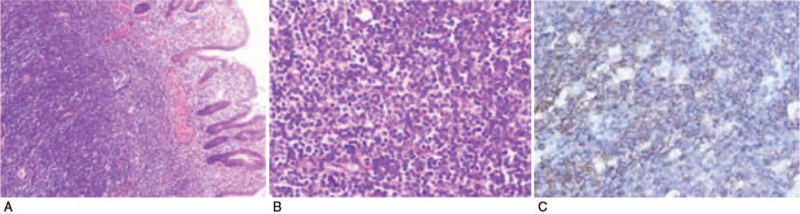
Intestinal wall with transmural lymphoma cell infiltrate. (A) H&E 20×; (B) H&E 200×; (C) Immunohistochemistry showing Vimentin (+), LCA (+), CD20 (+), CD79α (+), Bcl-2 (+), Bcl-6 (+), Kappa (+), Lambda (individual cells +), Background T cells CD3 (+), CD4 (+), CD5 (+), CD8 (+), CD10 (–), CD15 (–), follicular dendritic cells CD21 (+), CD23 (+), CD30 (–), CD38 (–), CD138 (–), CyclinD1 (–), ALK (–), mum-1 (–), CK broad (–), EMA (–), Ki-67 (10%+).

**Figure 2:**
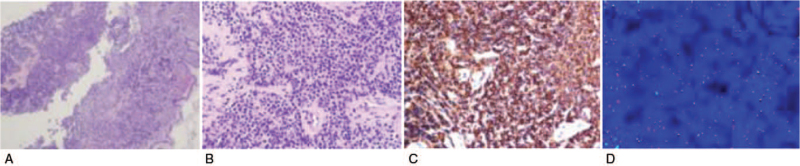
Gastric wall with lymphoma cell infiltrate. (A) H&E 20×; (B) H&E 200×; (C) Immunohistochemistry showing Vimentin (+), LCA (+), CD20 (+), CD79α (+), Bcl-2 (+), κ (+), λ (–), CD10 (–), mum-1 (+), Bcl-6 (–), CD3 (+), CD4 (+), CD5 (+), CD15 (–), CD30 (–), CD38 (–), CK broad (–), EMA (–), Ki-67 (10%+). (D) FISH showing green (MALT1(18q21)) signal, red (API2(11q22)) signal, and yellow fusion signal (API2/MALT1). FISH = fluorescence in situ hybridization.

A 48-year-old man presented to the hospital with abdominal pain, abdominal distention, nausea, and vomiting and stopped venting and defecation. Abdominal computerized tomography showed low intestinal obstruction, an annular high-density shadow, and a foreign body in stenosis. Partial resection of the small intestine near the tumor and anastomosis were performed immediately. Postoperative pathology revealed nodular marginal mucosa-associated lymphoma in the small intestine (Fig. 1). Bone marrow cytology showed no abnormal lymphocytes, and flow cytometry did not detect abnormal immunophenotypic lymphocytes in the submitted specimens. The bone marrow biopsy showed: CD20 minority (+), PAX5 individual (+), CD138 minority (+), cyclin-di (–), CD23(–), MPO multiple (+), AnnexinAL lymphocyte (–), Ki-67 (less than 5%+). In addition, computerized tomography examination of the head, neck, chest, and whole abdomen revealed no enlarged lymph nodes. Ultrasound gastroscopy suggested gastric lymphoma (Musshoff stage: IE), which now involved the muscular layer, and the API2/MALT1 fusion gene was positive (Fig. 2). On the basis of these results, the patient was diagnosed with MALT lymphoma Paris stage T4N2M1B0, Lugano stage-IV A.

The patient was re-examined after 2 courses of standard-dose R-CVP regimen chemotherapy (rituximab, cyclophosphamide, vincristine, and prednisone) combined with HP eradication therapy (lansoprazole, bismuth pectin, amoxicillin, and metronidazole). The ultrasound gastroscopy revealed thickened focal wall in the lower segment of the gastric body and the 5-layer structure of the stomach was absent, and there were low echo lesions of approximately 9.8 mm. We observed that the serous layer integrity was absent, there was external infiltration of the cavity and the pancreatic parenchyma echo was uniform; no dilatation of the main pancreatic duct, no dilatation of the bile duct. The gallbladder was normal, and there were multiple high-echo flares attached to the wall. An enlarged lymph node of about 21.7 × 15.9 mm in size was detected around the liver with a clear lymphatic hilum. Another round lymph node of about 5.9 × 5.4 mm was detected around the pancreas, with low echo and an unclear lymphatic hilum. Furthermore, no echo zone was detected between the intestines after examining an area of about 18.3 mm in width. No abnormal sound shadows were detected in the echo of the liver parenchyma. In addition, no enlarged lymph nodes were observed in the abdominal aortic root and mediastinum. The HP test was still positive. Therefore, the current treatment regimen was considered ineffective.

**Figure 3:**
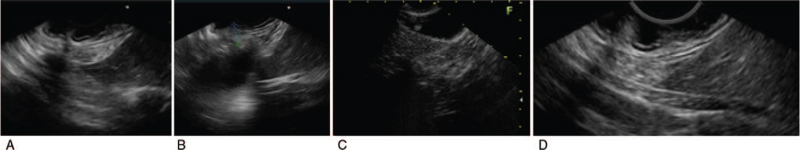
Ultrasound gastroscopy characteristics of MALT lymphoma. (A) Lymphoma involved the muscular layer muscular layer before chemotherapy; (B) The 5-layer structure of the stomach disappeared, and there were low echo lesions of about 9.8 mm after 2 courses of R-CVP; (C) Gastric wall at the thickest lesion in the lower segment of the gastric body decreased to 8.7 mm after 2 courses of lenalidomide-bendamustine treatment; (D) Pathological manifestations of the corresponding parts disappeared after 6 courses of lenalidomide-bendamustine treatment. MALT lymphoma = marginal zone B cell lymphoma of the mucosa-associated lymphoid tissue, R-CVP = rituximab, cyclophosphamide, vincristine, prednisone.

Therefore, R-CVP was interrupted, and treatment was switched to lenalidomide-bendamustine (lenalidomide 25 mg on days 1–21 and bendamustine 90 mg/m^2^ on days 1–2). This regimen was well tolerated, patient compliance was good, and there were no delays in administration and no infectious adverse events. After 2 courses of treatment, ultrasound gastroscopy showed that the thickness of the gastric wall at the thickest lesion in the lower segment of the gastric body had decreased to 8.7 mm. The lymph nodes around the liver shrank to 4.2 × 8.2 mm. After 6 courses of treatment, no pathological manifestations were observed in the corresponding parts (Fig. 3).

## 3 Discussion

API2-MALT1 translocation is a very frequent genetic event, reported in up to 25% of patients with gastric MALT lymphoma and 35% of patients with pulmonary MALT lymphoma, but is less common at other sites.^[5]^ The occurrence of API2-MALT1 has been associated with antibiotic resistance in HP-positive gastric MALT lymphoma. Previous reports showed that 66% of positive patients in a collective of 63 were unresponsive to HP eradication.^[3]^ Our patient also failed to respond to this treatment, as the patient did not respond to first-line chemotherapy. Currently, there is no consensus on the optimal treatment strategy for such cases.

MALT lymphoma can be characterized by a heterogenous infiltration consisting of small B-cells in the marginal zones of reactive B-cell follicles that extend into the interfollicular region as well as into the follicles (follicular colonization).^[1]^ Because the origin cell is assumed to be marginal zone B cells, there is an inextricable correlation with the plasma cell.^[6]^ Plasmacytic differentiation is present in approximately one-third of the gastric MALT lymphomas. Furthermore, there is a marked predominance of plasma cells in some MALT lymphomas. Because of this relationship and the proven impact of lenalidomide in different types of B-cell lymphomas, Leonard et al^[7]^ reported that lenalidomide can be an effective treatment for B-cell lymphomas. Furthermore, lenalidomide has been shown to improve the efficacy of rituximab in patients with recurrent indolent lymphoma with an acceptable safety profile.^[2]^ Kiesewetter et al^[2]^ reported that monotherapy with lenalidomide can induce objective responses in MALT-lymphomas. In their study, an overall response rate of 61% was observed. Therefore, we believe that lenalidomide is a good choice to control patients’ disease, but we are not sure whether lenalidomide is still effective in patients with relapsed/refractory MALT lymphoma. Therefore, to improve the therapeutic efficacy of our patient, we required more appropriate drugs that can be used in combination for relapsed/refractory MALT lymphoma.

Bendamustine is a cytotoxic compound that exhibits only partial cross-resistance to other alkylators, making it a possible treatment for relapsed/refractory patients.^[8]^ Kahl et al^[9]^ reported that single-agent bendamustine produced a high rate of objective responses with acceptable toxicity in patients with recurrent rituximab-refractory indolent B-cell lymphoma. Kiesewetter et al^[10]^ reported that bendamustine alone or in combination with rituximab showed clinical activity in several types of lymphomas, including MALT lymphomas, in the relapsed setting.

In view of these facts, it can be concluded that either lenalidomide or bendamustine may play an influential role in the monotherapy of MALT lymphoma. Therefore, we combined both treatments to control the patient's disease. In our study, this regimen was found to be safe and effective. No serious adverse events occurred during the treatment period. Ultrasound gastroscopy revealed that the tumor gradually shrank and eventually disappeared to complete remission. Therefore, we suggest that a combination of both drugs is applicable in some cases. As there is currently no unified treatment consensus for relapsed/refractory MALT lymphoma, this combination might be a potentially effective option for patients with refractory or relapsed MALT lymphoma after frontline therapy. However, further clinical trials are required to validate this approach.

## Acknowledgments

The authors thank the patient for his approval to publication.

## Author contributions

Conceptualization, design and writing: Zhencang Zhou, Yongqian Jia.

Clinical and research management: Zhencang Zhou, Pengqiang Wu, Fujue Wang, Huan Tao, Yingying Chen, Jie Gao, Dengke Chen.

All authors read and approved the final manuscript.

**Conceptualization:** Dengke Chen.

**Formal analysis:** Huan Tao.

**Investigation:** Huan Tao.

**Methodology:** Pengqiang Wu, Fujue Wang, Yingying Chen, Jie Gao.

**Resources:** Dengke Chen.

**Supervision:** Jie Gao.

**Visualization:** Yingying Chen.

**Writing – original draft:** Zhencang Zhou.

**Writing – review & editing:** Yongqian Jia.
